# Analysis of Influence of Coating Type on Friction Behaviour and Surface Topography of DC04/1.0338 Steel Sheet in Bending Under Tension Friction Test

**DOI:** 10.3390/ma17225650

**Published:** 2024-11-19

**Authors:** Tomasz Trzepieciński, Krzysztof Szwajka, Marek Szewczyk, Joanna Zielińska-Szwajka, Marek Barlak, Katarzyna Nowakowska-Langier, Sebastian Okrasa

**Affiliations:** 1 Department of Manufacturing Processes and Production Engineering, Faculty of Mechanical Engineering and Aeronautics, Rzeszów University of Technology, al. Powstańców Warszawy 8, 35-959 Rzeszów, Poland; 2 Department of Integrated Design and Tribology Systems, Faculty of Mechanics and Technology, Rzeszów University of Technology, ul. Kwiatkowskiego 4, 37-450 Stalowa Wola, Poland; kszwajka@prz.edu.pl (K.S.); m.szewczyk@prz.edu.pl (M.S.); 3 Department of Component Manufacturing and Production Organization, Faculty of Mechanics and Technology, Rzeszów University of Technology, ul. Kwiatkowskiego 4, 37-450 Stalowa Wola, Poland; j.zielinska@prz.edu.pl; 4 Plasma/Ion Beam Technology Division, Material Physics Department, National Centre for Nuclear Research Świerk, 7 Sołtana St., 05-400 Otwock, Poland; marek.barlak@ncbj.gov.pl (M.B.); katarzyna.nowakowska-langier@ncbj.gov.pl (K.N.-L.); sebastian.okrasa@ncbj.gov.pl (S.O.)

**Keywords:** coefficient of friction, coatings, friction, surface roughness, sheet metal forming

## Abstract

The working conditions of tools during plastic working operations are determined by, among other things, temperature, loads, loading method, and processing speed. In sheet metal forming processes, additionally, lubricant and tool surface roughness play a key role in changing the surface topography of the drawpieces. This article presents the results of friction analysis on the edge of the punch in a deep drawing process using the bending under tension test. A DC04 steel sheet was used as the test material. The influence of various types of titanium nitride and titanium coatings applied on the surface of countersamples made of 145Cr6 cold-work tool steel was tested by means of high-intensity plasma pulses, magnetron sputtering, and electron pulse irradiation. The influence of the type of tool coating on the evolution of the coefficient of friction, the change in the sheet surface topography, and the temperature in the contact zone is presented in this paper. An increase in the coefficient of friction with sample elongation was observed. Countersamples modified with protective coatings provided a more stable coefficient value during the entire friction test compared to dry friction conditions. The electron pulse irradiated countersample provided the highest stability of the coefficient of friction in the entire range of sample elongation until fracture. The skewness Ssk of the sheet metal tested against the coated countersamples was characterized by negative value, which indicates a plateau-like shape of their surface. The highest temperature in the contact zone during friction with all types of countersamples was observed for the uncoated countersample.

## 1. Introduction

Plastic working processes are among the fast and effective sheet metal processing technologies. Sheet metal forming methods ensure finished components in a short time compared to other technologies such as machining or casting. Manufacturing tools with increasingly better utility properties are the basis of every modern technology [1]. In the case of steel tools, the selection of the right steel grade, the correct course of technological operations of manufacturing, heat and thermo-chemical treatment, as well as the application of wear-resistant protective coatings are very important [2,3]. The cost of tools constitutes a significant share in the total production costs. Therefore, increasing the efficiency of tool functionality by increasing their durability, efficiency, and reliability is still one of the main indicators of increasing production efficiency [4,5].

The working conditions of tools depends on the processing temperature, contact pressure, loading method, and processing speed [6]. The problem of ensuring the required tool durability is therefore associated not only with the need to ensure the appropriate quality of tool materials and their heat treatment, but also with the selection of an appropriate protective coating and tool material [7]. In order to determine the area of application of tools and to select optimal processing parameters, it is necessary to know the working conditions of the tool [8]. By knowing these conditions, it is possible to determine the basic causes of its wear depending on the specific working conditions and to propose the most effective ways to increase its durability [9].

Applying wear-resistant protective coatings to tools is the fastest and usually the cheapest way to ensure appropriate friction conditions for tools in sheet metal forming [10,11]. New generation techniques involve modifying the surface layer of tools using hard layers of carbides and nitrides of elements such as titanium or vanadium. Two modern techniques of depositing hard layers on tools via chemical vapour deposition (CVD) and physical vapour deposition (PVD) are also widely used [12]. These technologies differ primarily in process parameters, temperature, and time, as well as mechanisms of layer formation. The CVD method involves the formation of hard layers of carbides or nitrides with the participation of carbide-forming elements [13]. The process can be carried out at atmospheric pressure in a gas atmosphere containing vapours of chemical compounds of the diffusing metal (usually in the form of a halide) and hydrocarbons, nitrogen, hydrogen, or an inert gas. Such materials include titanium carbides (TiC), titanium nitrides (TiN), aluminum oxides (Al_2_O_3_), and silicon nitrides (Si_3_N_4_). Multicomponent layers of the Ti(C, N), Ti(O, C, N) type or composite layers of the TiC + TiN type are also produced. These layers are applied to materials whose surfaces are subject to wear due to friction [14]. In the PVD method, layers are deposited by means of vapour crystallization from plasma, using physical phenomena such as metal or alloy evaporation, cathodic sputtering in a vacuum and ionization [15]. These vapours are deposited on a cold or heated substrate. The workpiece is then heated to a temperature of 200–600 °C, which allows coating of heat-treated steel tools without fear of a decrease in hardness. In order to eliminate this inconvenience, many PVD methods have been developed, which can be divided into vapour deposition, sputtering, and spraying methods. Layers obtained by the spraying method are characterized by very good adhesion. PVD methods are used to cover the surface of tools with titanium nitride TiN (less frequently with titanium carbide TiC) in order to obtain a multiple increase in their durability [16]. Currently, several dozen modifications of the PVD method are known [14]. Each modification uses different physical phenomena occurring at pressures in the range of 10-10^−5^ Pa [14]. The most important PVD varieties include activated reactive evaporation [17], bias activated reactive evaporation [18], pulse plasma method [19], magnetron sputtering [20], and ion beam deposition [21].

Laser and electron methods have found particularly wide practical applications. The advantage of laser and electron methods over conventional methods results mainly from [22] short heating process duration, limitation of heating to the strictly near-surface layer of the material, and very high cooling rate of the material. Among the considered laser processing technologies, the following should be mentioned: laser heat treatment, laser remelting, laser alloying/surfacing, laser additive manufacturing, laser-assisted chemical vapour deposition, and laser-assisted physical vapour deposition [23,24]. Reactive magnetron sputtering consists of sputtering the material by gas ions generated in the area between the plasma and the workpiece. Magnetron sputtering physical vapour deposition allows the deposition of many types of materials, including ceramics and metals [25]. Magnetron sputtering is an improved version of the PVD method, in which the basic sputtering process was limited, among others, by low ionization efficiency in the plasma. Magnetron sputtering uses the fact that the magnetic field is directed parallel to the target surface, which reduces the secondary movement of electrons near the target [26]. Thanks to this method, it is possible to produce hard, abrasion-resistant coatings characterized by a low coefficient of friction, as well as corrosion-resistant coatings and coatings with specific optical and electrical properties [20]. Electron-pulse irradiation ensures super high rates of heating of the surface layer to high temperatures and equally high rates of its cooling due to the removal of heat into the internal volume of the alloy [27,28].

The sheet forming process may cause premature damage to the tool surface due to the high forming pressure and excessive wear of the workpiece surface [29]. Hard coatings like CrN, TiAlN, TiN, etc. have already outperformed traditional cold-work steel tools in the majority of forming operations [30,31]. The most dominant wear mechanisms, found in sheet metal forming operations, are adhesive and abrasive wear [30]. Due to the mechanical loads of stamping dies, the proper selection of substrate is crucial for the proper performance of the coated tools. The surface roughness of sheet metal-forming tools has a decisive influence on the lubrication conditions [32] and thus on the final surface roughness of the components [33,34]. Additionally, plastic deformation changes the friction conditions as a result of the change in the sheet metal surface topography [35] and changes the sheet metal properties as a result of the work hardening phenomenon [36].

In this article, the influence of different types of coatings produced by the high-intensity plasma pulses, the magnetron sputtering and electron pulse irradiation methods on friction conditions, surface topography changes, and the temperature in the contact zone in sheet metal forming process was investigated. To the best of the authors’ knowledge, similar studies on friction in punch edge sheet metal forming have not been conducted so far. The experimental studies used a tester manufactured by the authors to perform the bending under tension (BUT) friction test.

## 2. Materials and Methods

### 2.1. Test Material

**Figure 1:**
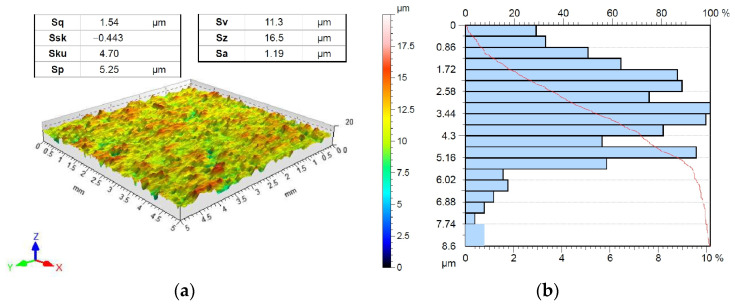
(**a**) Topography and (**b**) bearing area curve of DC01 steel sheet surface.

The test material was 0.8 mm-thick DC01 (1.0330) steel sheets. This steel is used for simple forming operations such as drawing, beading, and bending. According to EN, 10130 standard DC01 steel sheet contains (max.) 0.12 wt.% of carbon, 0.60 wt.% manganese, and 0.045 wt.% each of sulphur and phosphorus. The basic mechanical properties of the test material were determined in a uniaxial tensile test according to EN ISO 6892-1 [37] standard. In the experiments, the Zwick/Roell Z100 machine was used. Three samples were cut along and across the sheet in the rolling direction and the average values of the mechanical parameters were determined. This resulted in a yield stress of 163.5 MPa, an ultimate tensile strength of 285.4 MPa, and an elongation of 25.2%. The surface roughness of the sheets and countersamples was measured using a T8000RC stationary profilometer manufactured by Jenoptik AG. The values of the selected surface roughness parameters were determined based on the ISO 25178-2 [38] standard. The profilometer is characterized by a measuring range roughness of ±300 μm. The vertical and horizontal resolution of this profilometer is 0.05 μm and 0.1 μm, respectively. The surface roughness of strip samples was measured at the middle of their width, halfway up. The measurement parameters include the following: cut off = 0.8 mm, feed rate = 0.5 mm/s, measuring tip radius = 5 μm, and dimensions of measured surface = 5 × 5 mm. Figure 1a shows a view of the topography and basic 3D surface roughness parameters of the sheet surface in the as-received state. Figure 1b shows the bearing area curve describing the surface texture of the sheet surface. Samples for friction testing were cut along the sheet rolling direction.

### 2.2. Friction Testing

**Figure 2:**
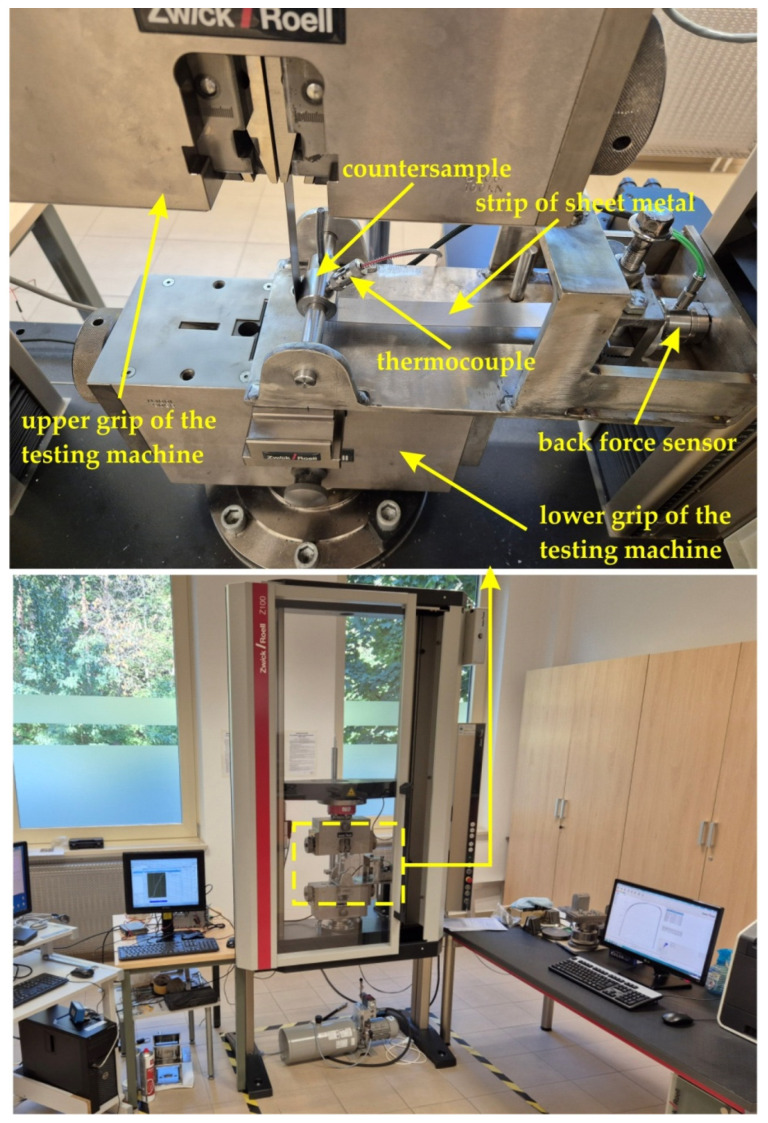
Test stand.

**Figure 3:**
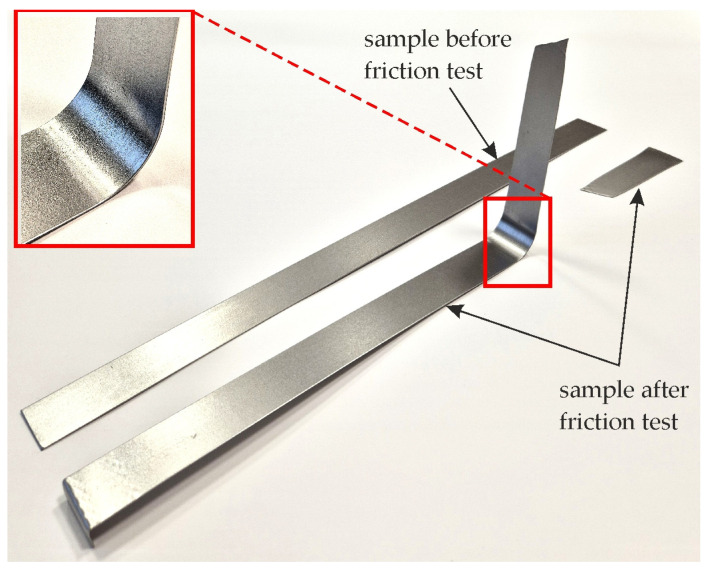
View of samples before and after friction process.

**Figure 4:**
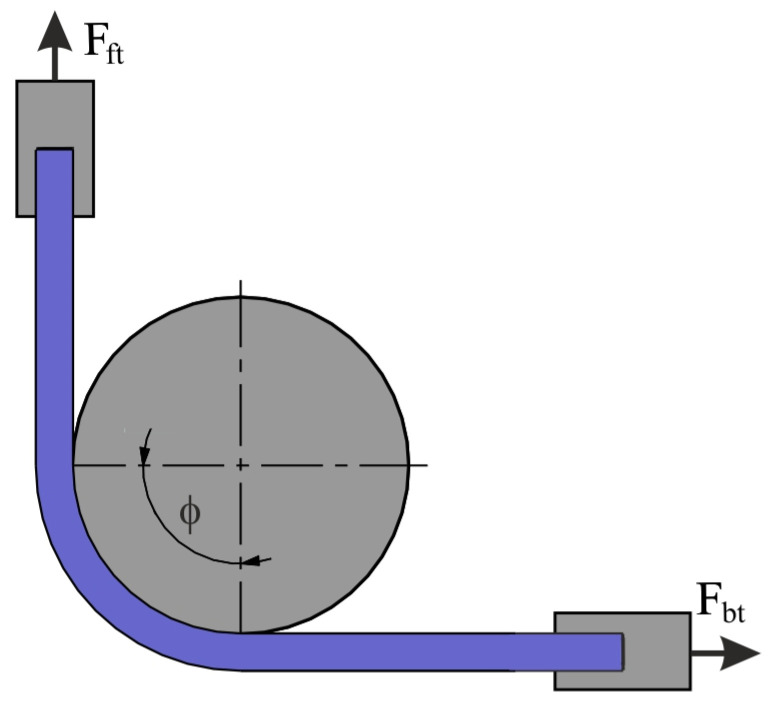
A schematic of the BUT test.

The test was carried out using a special tester, which is mounted in the lower gripper of the uniaxial tensile testing machine (Figure 2). The test material consisted of 400 mm long and 20 mm wide strips of sheet metal (Figure 3). One end of the sheet metal was mounted in the holder of the device integrated with the back force sensor Kistler type 9345B (Kistler, Winterthur, Switzerland), which measures the back tension force (BTF) F_bt_ (Figure 4). The other end of the sample was placed in the gripper of the uniaxial tensile testing machine, which moved at a speed of 0.03 m/min. The front tension force (FTF) F_ft_ (Figure 4) was recorded using this machine. Both BTF and FTF were recorded with a frequency of 100 Hz. The BTF was recorded via a Kistler type 5073 (Kistler, Winterthur, Switzerland) charge amplifier. A strip sample was subjected to a BUT test until fracture (Figure 3).

The countersamples were drilled with holes in which a resistance temperature detector 745691-02 supplied by National Instruments (Austin, TX, USA) (NI) was placed. The temperature in the contact zone was recorded using the cDAQ-9132 station and the 9216 temperature measurement module (both NI). The value of the coefficient of friction was determined as follows [39].

(1) $$\mu =\frac{2}{\pi }ln\frac{F_{ft}}{F_{bt}}$$

The cylindrical countersamples were made of 145Cr6 cold work tool steel. Three types of anti-wear coatings were applied to the surfaces of countersamples as shown in Table 1. For comparison, uncoated specimens were also used.

Ti- and TiN-coated tools reduce abrasion and adhesive wear as well as reduce friction. Its use in plastic working tools improves tool life up to three times relative to uncoated tools [40]. As a reference, a modification of the surface layer by titanium was used. Such layers are used to increase the wear resistance of heavily loaded machine components [41,42]. Additionally, for comparison purposes, tests were performed on uncoated countersamples.

Before the friction process, all strip samples and countersamples were cleaned with acetone. The tests were carried out under typical conditions that exist in sheet metal forming: dry friction conditions and lubrication conditions. For the lubrication conditions, two deep drawing oils with significantly different kinematic viscosities were selected: S300 and S100+. The research assumed that the sheets were formed in cold forming conditions (at room temperature). The kinematic viscosity at the test temperature (20 °C) of the oils used ranged from 360 mm^2^/s (oil S100+) to 1135 mm^2^/s (oil S300). The oils used are intended for cold forming conditions.

**Figure 5:**
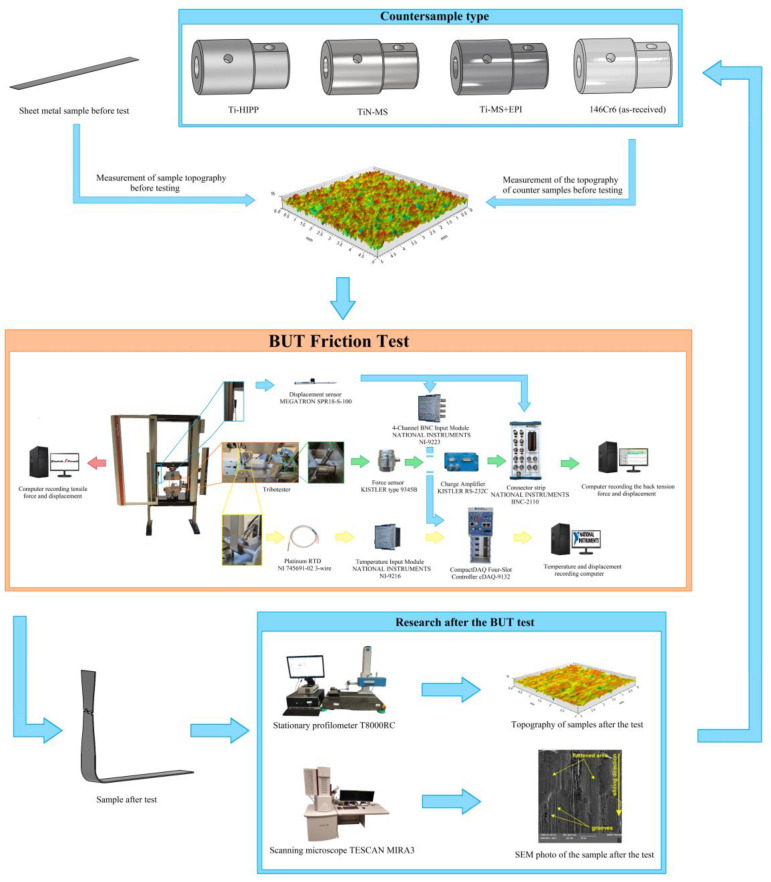
Flow chart for experimental investigations.

Figure 5 shows a flow chart that more clearly describes the investigation steps that were performed in this paper.

### 2.3. Preparation of Countersamples

**Figure 6:**
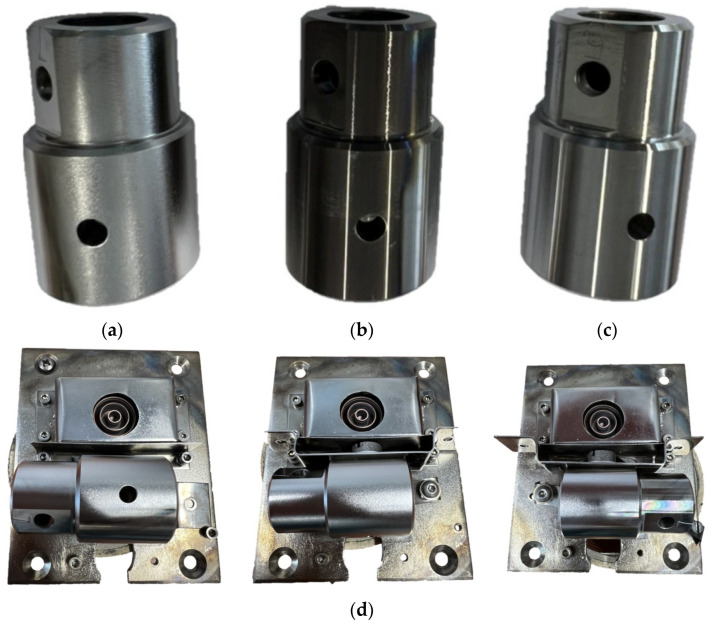
The general view of the modified countersamples: (**a**) Ti-HIPP, (**b**) TiN-MS, (**c**) Ti-MS+EPI, and (**d**) three consecutive (from left to right) orientations of countersample during modification using electron gun.

The surface modification of the countersamples was performed using the methods exploited at the National Centre for Nuclear Research Świerk (Otwock, Poland). Three 145Cr6 steel countersamples (Figure 6a–c) were modified using the high-intensity plasma pulses, magnetron sputtering, and electron pulse irradiation (Table 2). The countersamples were modified in 2–3 steps, due to their cylindrical shape. The modification in the second and third step was performed after the rotation of the countersample (Figure 6d).

#### 2.3.1. Ti-HIPP Countersamples

**Figure 7:**
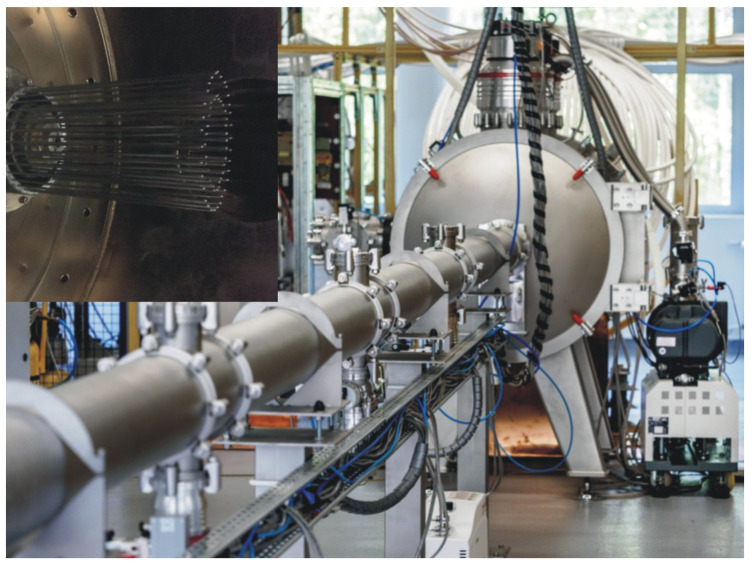
RPI-IBIS device for high-energy plasma generation + view of coaxial rod plasma accelerator with titanium electrodes.

The method of high-intensity plasma pulses, described in details in [43], enables the modification of the surface of materials with plasma pulses with an energy density of several dozen J/cm^2^ and a duration of microseconds, using a rod plasma injector (RPI) type generator (IBJ, Świerk, Poland) (Figure 7). Depending on the operating mode (Pulse Implantation Doping (PID) or Deposition by Pulse Erosion (DPE)), it enables the supply of energy or energy and mass to the system. This enables both the heating of the surface after other processing [44] and its modification [45]. With the appropriate selection of the process parameter values, the modified area is not a layer and therefore there is no adhesion problem as in the case of the above-mentioned PVD method. Additionally, the amount of the introduced dopant and the width of the modified area is greater than in the case of, for example, ion implantation [46].

The rod plasma injector type generators of intense plasma pulses are the devices enabling melting of the surface of a material and the introduction of atoms of a dopant to the melted area. The total energy density on the exposed substrate is a function of the main capacitor bank voltage and its capacitance. It is also dependent on the distance between the tips of the electrodes and the substrate surface along the axis of the plasma stream. The surface energy loads were derived from temperature differences in the specially designed thermocouple matrix probe, situated in the axis of the plasma stream. Since the sample holders covered most of the matrix measurement points, the energy distribution was measured and the repeatability of the process was checked before the installation of the sample. Throughout the conducted experiments, the target surface was maintained at room temperature before each plasma pulse.

The main parameters of the high-intensity plasma pulses process are presented in Table 2.

**Table 2:** The parameters of the high-intensity plasma pulses.

Parameter	Value
material of electrodes	Ti
working gas	He
pulse duration	5 µs
number of plasma pulses	2 × 3
average pulse energy density	4 J/cm^2^
distance between electrodes and modified surface	25 cm

#### 2.3.2. TiN-MS Countersamples

**Figure 8:**
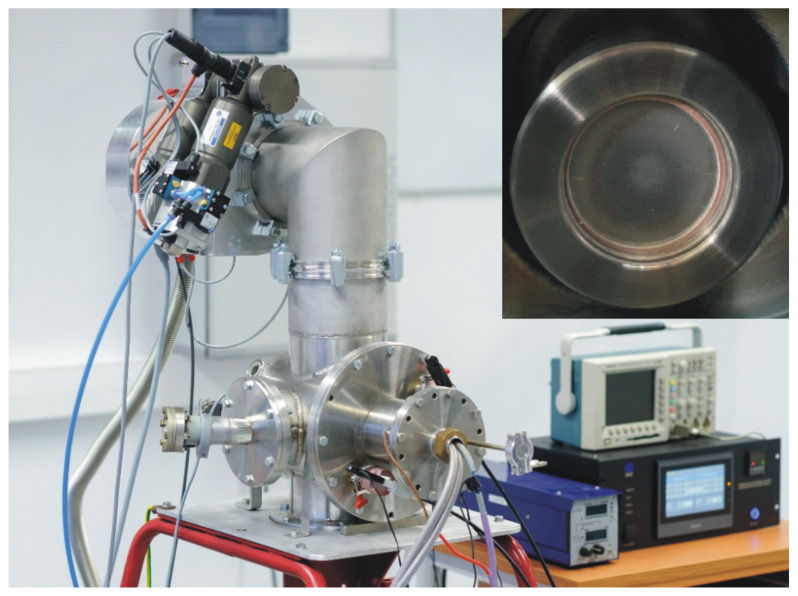
The device for layer deposition by the pulsed magnetron sputtering method + view of the magnetron inside the chamber with a titanium target.

The WMK 50 (IMT, Wroclaw University of Technology, Wrocław, Poland) unbalanced magnetron (Figure 8) was used in the synthesis by the pulsed magnetron sputtering (PMS) method.

The method of pulsed magnetron sputtering was used for Ti and TiN layers deposition due to the high control over the process parameters, enabling the production of layers with the desired structure as well as chemical and phase composition. The advantage of this technology is the low temperature and pressure of the process, which allows for the synthesis of materials sensitive to high temperatures [47]. One of its main benefits is its wide range of applications, both in industry and in scientific research, which facilitates scaling to production processes [48]. This method also enables the execution of reactive processes, involving the creation of new chemical compounds in plasma, which provides additional forms of energy [49]. These tools are not offered by traditional techniques without plasma assistance, such as chemical reactions [50], chemical vapour deposition (CVD) [51], and physical vapour deposition (PVD) [52].

Magnetron sputtering is a PVD method used for the deposition of thin film or coatings. The pulse magnetron sputtering used here is a variation in the deposition technique in which the power is applied to the target in pulses of low duty cycle (<10%) and frequency (<10 kHz).

The main parameters of the processes were as follows:

- Material of the target: Ti (thickness of 6 mm),
- Working gas: Ar (pressure of 0.7 Pa)
- Total pressure of Ar+N_2_: 0.742 Pa,
- Effective power: 2500 W,
- Modulation frequency: 1000 Hz,
- Distance between the target and modified surface: 8 cm,
- Processing time: 2 × 35 min,
- Estimated temperature on the stage: 120 °C = cold substrate during the synthesis process,
- Thickness of the deposited layer: 800 nm.

#### 2.3.3. Ti-MS+EPI Countersamples

**Figure 9:**
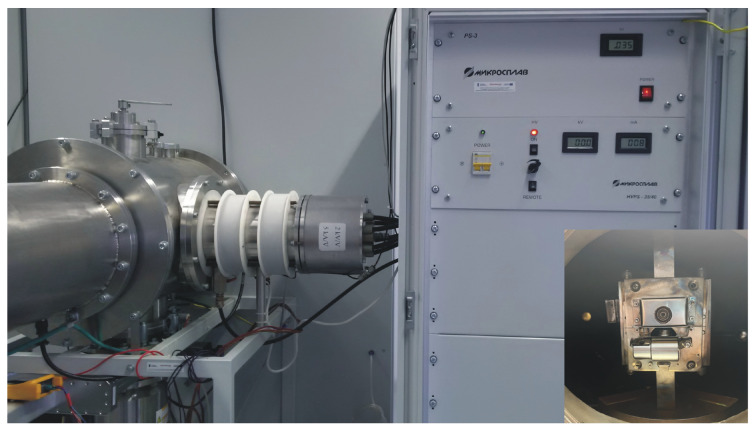
Electron gun device + countersample inside vacuum chamber of electron gun.

The electron irradiation (EI) processes were performed using the “RITM-2M” research device (Figure 9) manufactured by Microsplav OOO under the licence of the Institute of High Current Electronics, Tomsk, Russia, in the form of a generator of non-relativistic, high-current (up to 25 kA) pulsed electron beams of microsecond duration (2 µs). This generates a quite homogeneous wide aperture electron footprint up to 10 cm in diameter, determined by the studies in the field of physics of the interaction of high-energy beams with a solid [53,54]. The electron energy density was determined by a copper calorimeter with a thermistor temperature measurement, attached to the sample holder close to the sample.

This method allows the previously deposited layer to be embedded in the substrate, without additional dopants, similarly to high-intensity plasma pulses in the PID mode. Another advantage is that the high dose rate requires very short irradiation time [55]. On the other hand, the high dose rate requires high temperatures that must be closely controlled [55], and electron irradiation can cause surface heating [56].

The modification was provided in 2 stages due to the relatively low adhesion of the Ti layer to the steel substrate. In the first stage, the above described (Section 2.3.2) magnetron sputtering method was applied. The main parameters of the processes were as follows:

- Material of the target: Ti (thickness of 6 mm),
- Working gas: Ar (pressure of 0.7 Pa),
- Effective power: 2500 W,
- Modulation frequency: 1000 Hz,
- Distance between the target and modified surface: 8 cm,
- Processing time: 2 × 15 min,
- Estimated temperature of the modified countersample: 120 °C,
- Thickness of the deposited layer: 800 nm.

In the second stage, the countersample was modified using a research device in the form of a generator of non-relativistic, high-current (up to 25 kA) pulsed electron beams of microsecond duration (simply named as electron gun—Figure 8), which generate a quite homogeneous wide aperture electron footprint up to 10 cm in diameter, determined by the studies in the field of physics of the interaction of high-energy beams with a solid.

The electron energy density was determined by a copper calorimeter with a thermistor temperature measurement, attached to the sample holder close to the sample.

The main parameters of the processes were as follows:

- Working gas: Ar of 99.999% purity,
- Number of pulses: 3 × 1,
- Pulse duration: 2 µs,
- Acceleration of the voltage in peak/electron energy: 25 kV/25 keV,
- Pulse energy density: 2.44, 3.18, 3.31 J/cm^2^.

### 2.4. Characteristics of Countersamples

**Figure 10:**
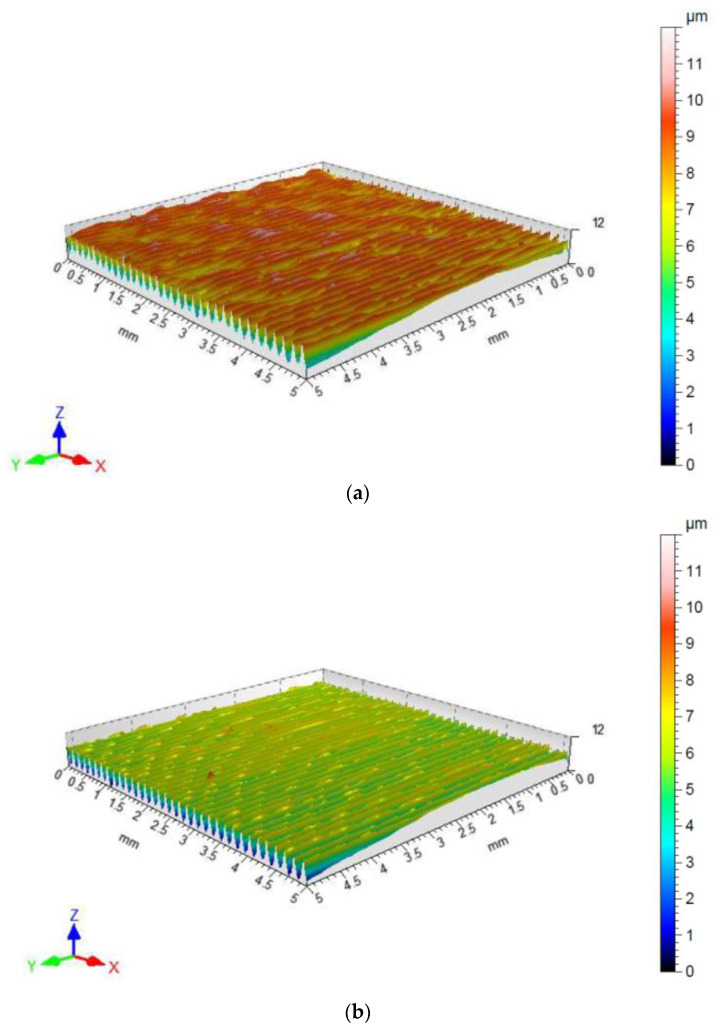

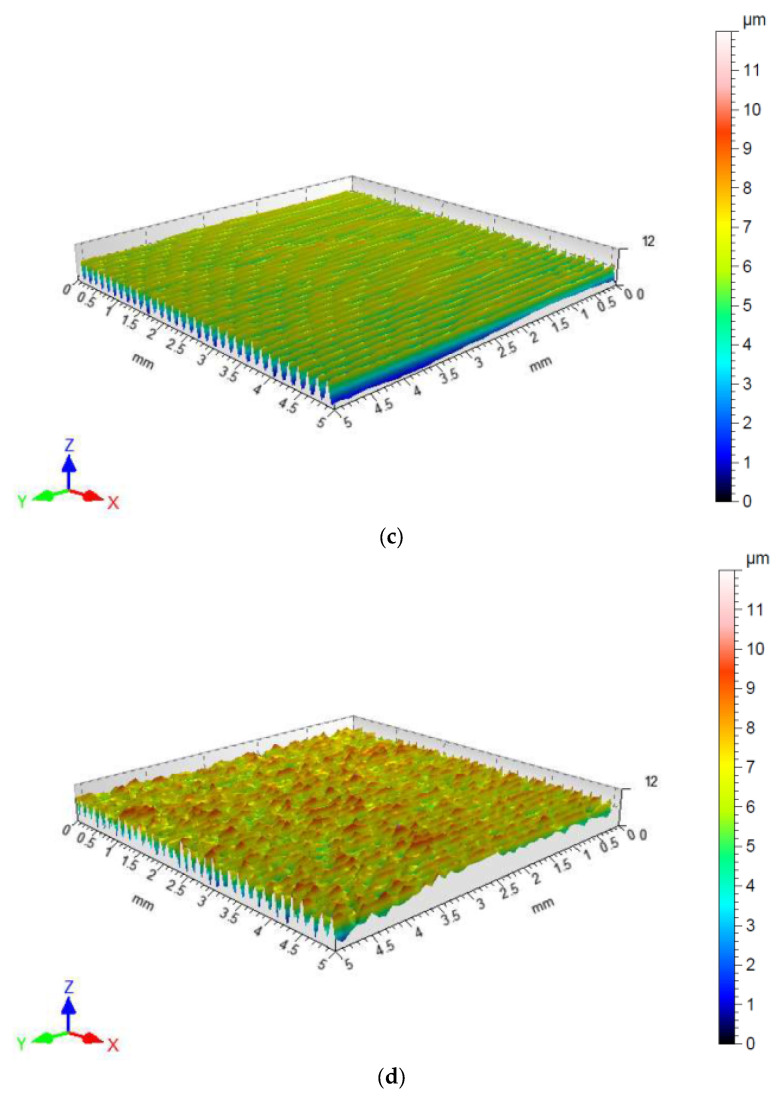
Surface topography of cylindrical countersamples: (**a**) as-received state, (**b**) Ti-HIPP, (**c**) TiN-MS, and (**d**) Ti-MS+EPI.

**Figure 11:**
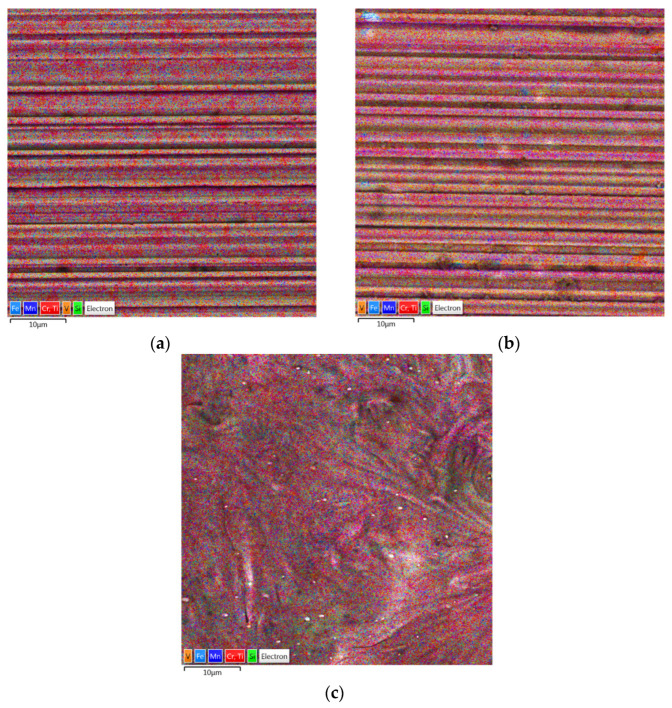
EDS layered images of countersamples: (**a**) Ti-HIPP, (**b**) TiN-MS, and (**c**) Ti-MS+EPI.

**Figure 12:**
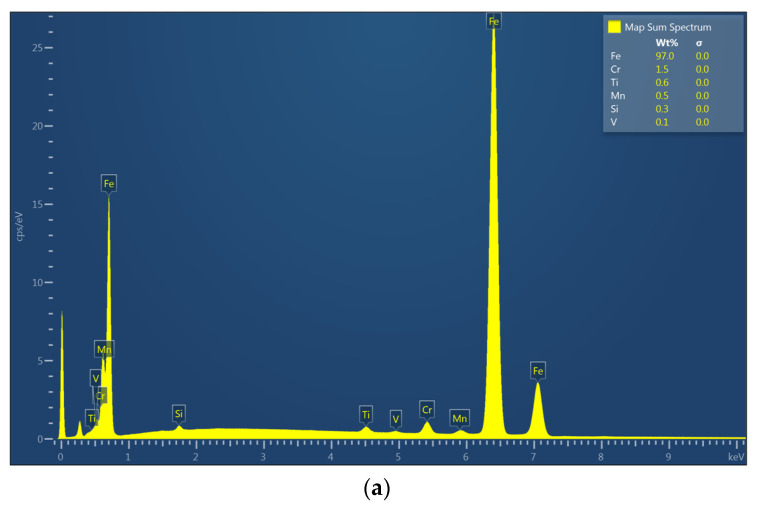

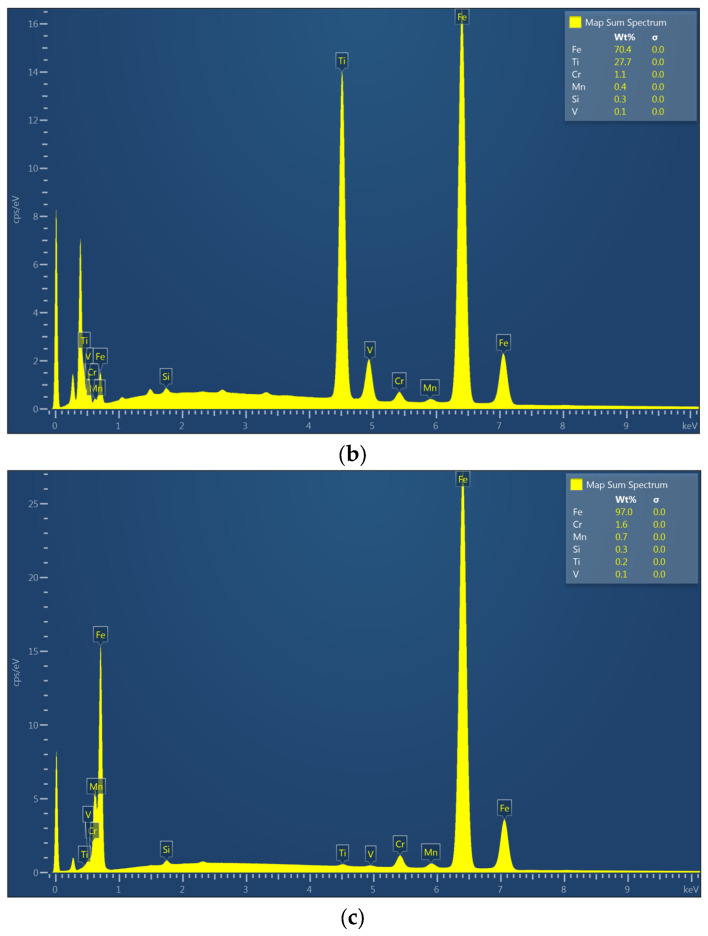
The EDS spectrum of the following coatings: (**a**) Ti-HIPP, (**b**) TiN-MS, and (**c**) Ti-MS+EPI.

Surface topographies of the countersamples and values of the selected 3D surface roughness parameters are presented in Figure 10 and Table 3, respectively. The surface morphologies of the countersamples were examined by scanning electron microscopy and energy dispersive X-ray spectrometry (Oxford Instruments, Abingdon, Great Britain). EDS layered images of the countersamples (Figure 11) confirm the presence of a u, niform layer containing mainly chromium and titanium on the surface of the countersamples. The EDS spectra of the countersample surfaces are shown in Figure 12. On the surface of TiN-MS countersamples, titanium content equal to 27.7 wt.% occurs. In the case of the other countersamples, titanium content does not exceed 0.2 and 0.6 wt.% in the case of Ti-MS+EPI and Ti-HIPP, respectively.

**Figure 13:**
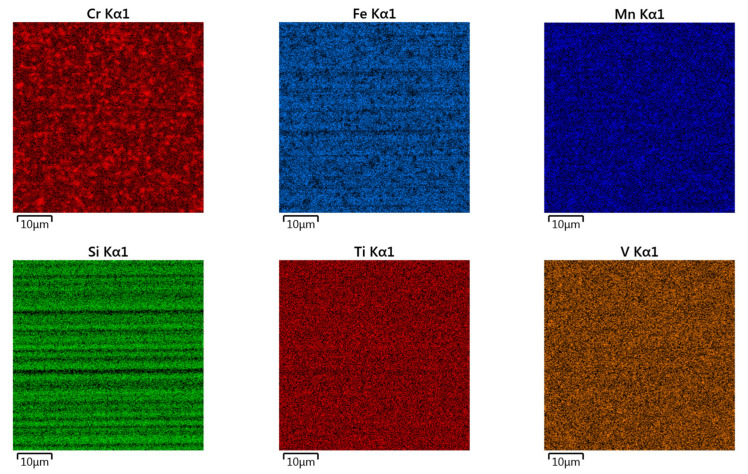
EDS elemental mapping of Ti-HIPP coating.

**Figure 14:**
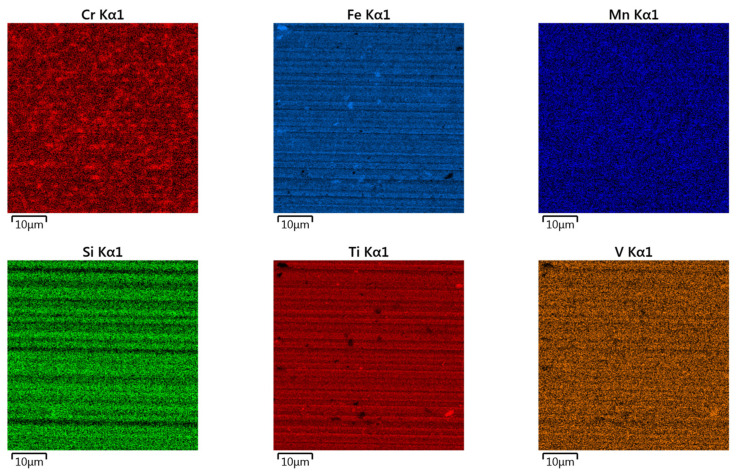
EDS elemental mapping of TiN-MS coating.

**Figure 15:**
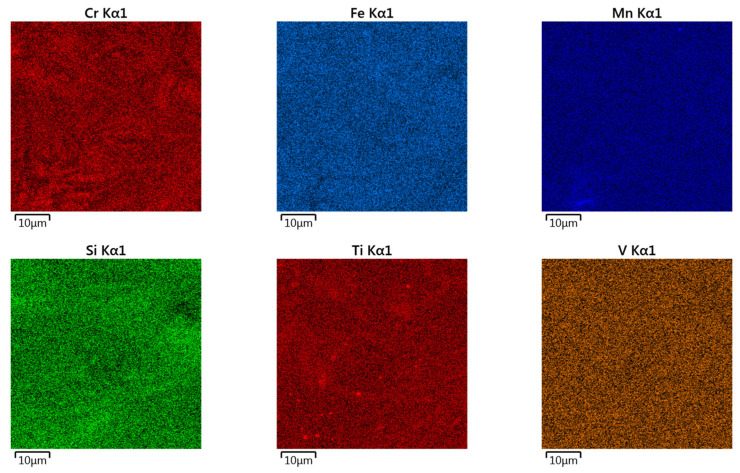
EDS elemental mapping of Ti-MS+EPI coating.

Additionally, Figure 13, Figure 14 and Figure 15 present the EDS (Energy Dispersive Spectroscopy) results for each element separately. This would provide a clearer analysis of the elemental composition of the coatings and facilitate a better understanding of the distribution and concentration of each element.

## 3. Results and Discussion

### 3.1. Coefficient of Friction

**Figure 16:**
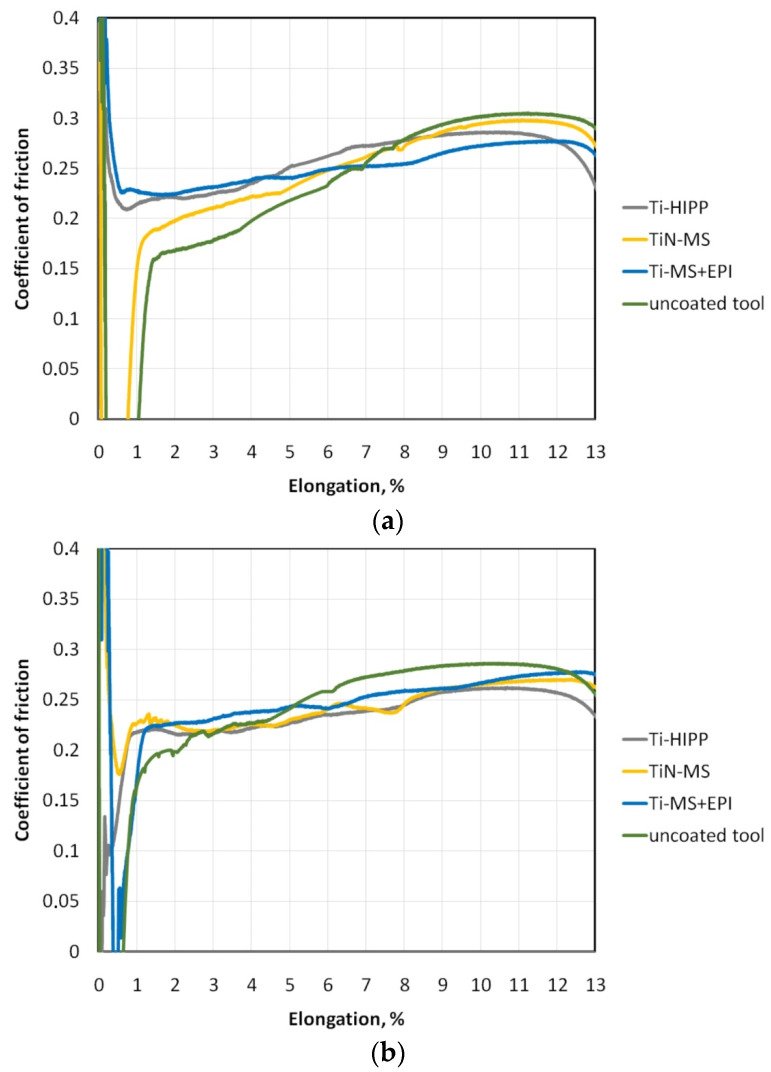

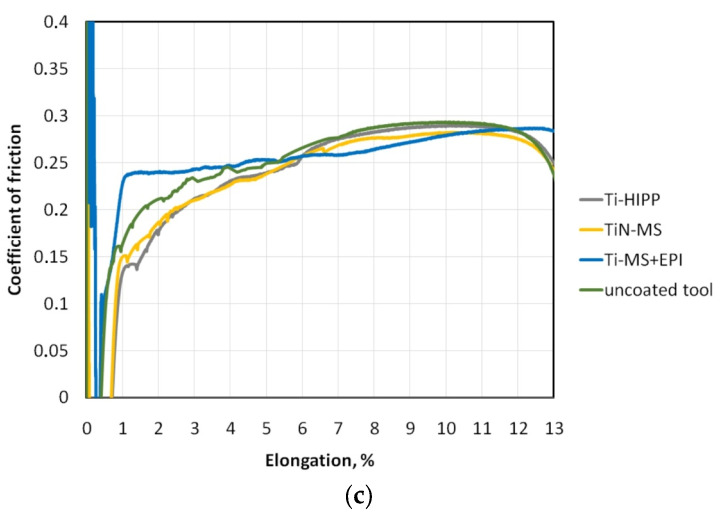
Effect of sample elongation on coefficient of friction measured under (a) dry friction and lubricated conditions using (b) S100+ oil and (c) S300 oil.

Figure 16 shows the variation in the coefficient of the friction value during the bending under tension test. A clear tendency to increase the friction coefficient value with sample elongation can be observed. Stretching the sample causes a simultaneous change in the topography of the sheet surface and a change in its properties as a result of the work hardening phenomenon. The tested DC01 steel sheet is characterized by relatively good formability and moderate elongation (A = 25.2%) in uniaxial tension test conditions. Bending the sample with simultaneous stretching in the BUT test under friction conditions reduced the elongation at the break by about half (Figure 16).

In the case of dry friction conditions (Figure 16a) and the uncoated countersample, the lowest friction coefficient value was recorded in the initial phase of the friction test, and after exceeding the sample elongation of about 8%, the uncoated countersample produced the highest coefficient of friction among all tested types of countersamples. The countersamples modified with protective coatings (Ti-HIPP, TiN-MS, and Ti-MS+EPI) provided a more stable friction coefficient value during the entire friction test. During friction with the Ti-coated countersample after magnetron sputtering and electron pulse irradiation (Ti-MS+EPI), the friction coefficient value varied in a narrow range between μ = 0.22 and μ = 0.27. Small differences in the coefficient of friction variation during the BUT test indicate a better ability of the coating to reduce the coefficient of friction. In the final stage of the test, the friction coefficient value determined as the ratio between front tension force and back tension force (Equation (1)) decreases.

The countersamples modified with Ti and TiN protective coatings were characterized by an average roughness Sa lower by about 3.2–8.6% compared to the uncoated countersample made of 145Cr6 steel in the as-received state (Sa = 1.87 μm). In the conditions of lubrication with S100+ oil, after exceeding the sample elongation of about 5%, the most unfavourable friction conditions occurred for the uncoated countersample (Figure 16b). At lower values of sample elongation in the conditions of lubrication with this oil, the friction coefficient values for all types of countersamples are similar to μ = 0.2–0.24. Ignoring the initial unstabilized range of friction coefficient changes resulting from the sample tensioning between the grips, it should be stated that the friction coefficient value for the coated countersamples varied between 0.22 and 0.27.

The S300 oil was characterized by a viscosity value approximately three times higher than the viscosity of the S100+ oil. Despite this, by comparing the results of the friction coefficient changes for the tests carried out under lubricated conditions, it can be seen that the S300 oil (Figure 16c) in the range of elongation between 3% and 12% provided similar friction conditions as the S100+ oil (Figure 16b).

In all the analyzed friction conditions, the Ti-MS+EPI countersample provided the highest stability of the coefficient of friction in the entire range of sample elongation until fracture. It should be noted that the contact pressure value increased during the entire test due to the increasing resistance to sample deformation caused by work hardening. In high-pressure conditions, the applied lubricant was not able to effectively reduce the friction coefficient value. Effective lubrication is hindered by the continuous evolution of the surface topography resulting from the stretching of the strip samples and the inability to create closed oil pockets that are able to generate hydrostatic pressure in the surface valleys [57,58]. The similar value of the coefficient of friction confirms that during the BUT test, the dominant friction phenomenon was the mechanical interaction of the asperities of the bodies in contact. The oil was not able to effectively reduce resistance to friction in the conditions of strongly variable surface topography.

### 3.2. Surface Roughness

**Figure 17:**
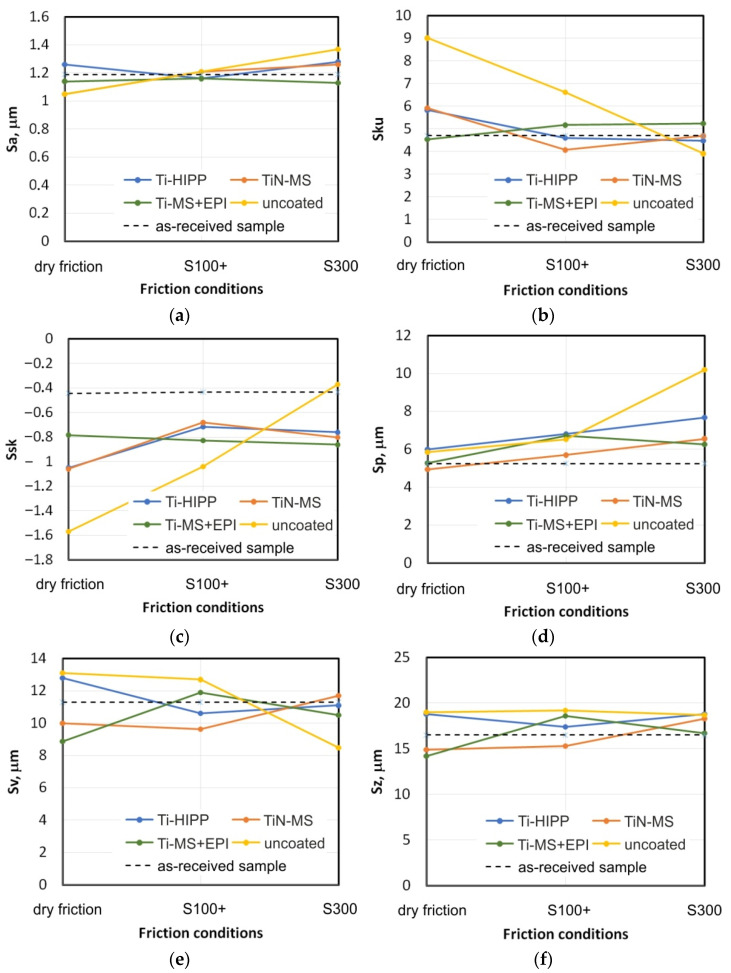
Effect of friction conditions on selected surface roughness parameters: (a) Sa, (b) Sku, (c) Ssk, (d) Sp, (e) Sv, and (f) Sz.

Figure 17 shows the change in the selected sheet surface roughness parameters measured on samples in the contact zone after the friction process. The average roughness Sa (Figure 17a) is commonly used to characterize the surface of sheets in sheet metal forming [59,60,61]. During dry friction, the uncoated countersamples caused the largest change in the average roughness. In the case of the coated countersamples, this change was smaller and ranged between 4.2% (Ti-MS+EPI) and 11.8% (Ti-HIPP, TiN-MS). In the conditions of S100+ oil lubrication, only titanium coated countersamples (Ti-HIPP and Ti-MS+EPI) caused a decrease in the average roughness Sa. In the friction conditions of the remaining countersamples, the Sa parameter increased. It should be noted that in the conditions of lubrication with S300 oil, a significantly greater change in topography was observed than during friction with S100+ oil. The uncoated sample led to the largest change in the Sa parameter (about 7.6%). Only the Ti-MS+EPI countersample provided a reduction in the average roughness of samples in all friction conditions, and the percentage change value of this parameter was almost the same for all friction conditions.

**Figure 18:**
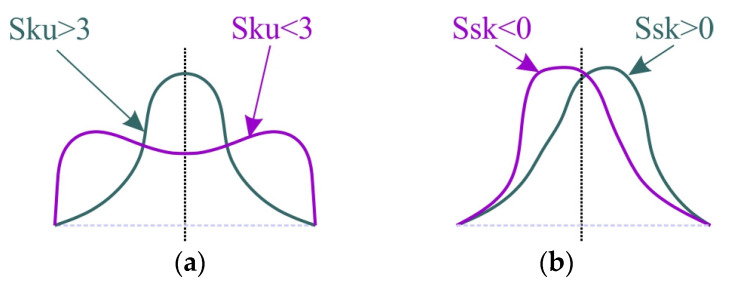
Interpretation of parameters (**a**) Sku and (**b**) Ssk.

Kurtosis Sku is a measure of the asymmetry of the profile about its mean line. This parameter is also called a measure of the flatness of the profile height distribution. If Sku is equal to 3, it means a Gaussian distribution. A kurtosis value greater than 3 means a distribution centred around the mean line (Figure 18a). On the other hand, at a kurtosis value below 3, the z-value distribution graph is flattened [62]. In the lubricated conditions with Ti- and TiN coated countersamples, a surface with kurtosis between 4 and 5.1 was observed. It should be noted that during friction with the Ti-MS+EPI countersamples, the kurtosis is most similar to the sheet metal surface in the as-received state.

The uncoated countersample caused the most significant change in kurtosis value with respect to the as-received surface. During dry friction and lubrication with S100+ oil, the kurtosis value increased by about 90.7% and 40.8%, respectively. Only during friction with S300 oil, the kurtosis decreased by about 16.8%.

**Figure 19:**
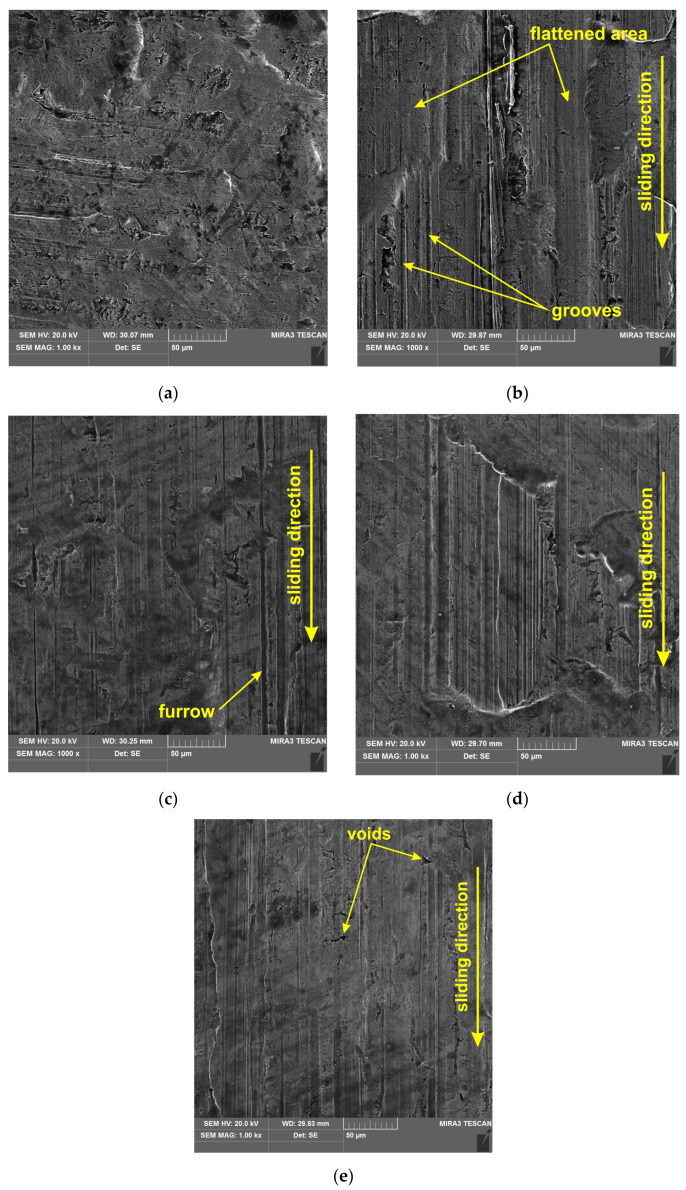
The SEM micrographs of the surface of (**a**) the DC01 sheet in the as-received state and after friction tests under the following conditions: (**b**) Ti-HIPP, dry friction; (**c**) Ti-HIPP, lubrication with S100+ oil; (**d**) TiN-MS, lubrication with S100+ oil; and Ti-MS+EPI, lubrication with S300 oil.

**Figure 20:**
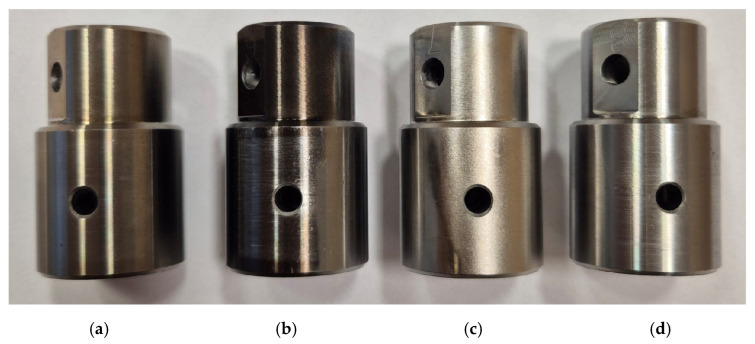
View of working surfaces of (**a**) uncoated countersample and coated countersamples: (**b**) Ti-HIPP, (**c**) TiN-MS, and (**d**) Ti-MS+EPI.

The skewness parameter is sensitive to the occurrence of local elevations or valleys on the surface. The skewness Ssk, in the case of the tests involving coated countersamples, has a negative value, which indicates a plateau-like shape of their surface (Figure 18b). The lower Ssk value, the more flattened the surface and the more rounded the peaks. Similarly to the Sku parameter, the uncoated countersample triggered the largest changes in the Ssk parameter depending on the friction conditions (Figure 17c). Only friction with S300 oil and the uncoated countersample caused a slight increase of 16.5% (from −0.43 to −0.37) in skewness in relation to the as-received surface. In these conditions, the surface could have been ploughed, which may be confirmed by the high value of the Sp parameter corresponding to this change (Figure 17d). The distribution of the skewness values confirms the flattening of the peaks of the asperities as a result of the friction process. Figure 19 presents a comparison of SEM micrographs of sheet metal in the as-received state and selected surfaces of strip samples after the friction process. Large areas of flattening of surface asperities and grooves can be observed. Flattening of the summits of sheet metal asperities is an inherent part of sheet metal-forming processes. The sheet material is much softer than the tool material, which must be highly reliable. The process of flattening the asperities in the BUT test is intensified by stretching the samples. It should also be noted that the sheet metal surface in the as-received state is not perfectly flat and is characterized by an uneven surface profile with voids and microcracks resulting from the manufacturing (rolling) process. The largest elevations in the first stage undergo plastic deformation during the friction process. The distinct directional topography of the sheet surface after the friction process is the result of cooperation of the asperities of the sheet metal surface with the surface of the much harder countersample. Grooves and furrows can be the source of the interaction of the highest peaks of the tool surface asperities and, additionally, they can be formed as a result of the impact of work hardened sheet material particles stuck to the surface of the countersample. Figure 20 shows a view of the working surfaces of the countersamples. No obvious signs of wear were observed.

Similarly to the Sa and Sku parameters, the friction with Ti-MS+EPI countersample provided the most stable Sku value for all friction conditions. This value varied from −0.78 for dry friction to −0.86 for S300 oil lubrication.

Sp is the height of the highest peak within the defined area. In general, the application of oils resulted in an increase in the Sp parameter value (Figure 17d). The exception is friction with the TiN-MS countersample. However, the recorded decrease in the Sp parameter was about 6%. Lubrication conditions facilitate the movement of the sheet metal over the friction surface, which at the same time facilitates the change in the surface topography as a result of sheet metal stretching.

The analysis of the maximum pit height Sv changes (Figure 17e) is difficult to interpret. Each of the countersamples presents a different character of the Sp parameter changes depending on the friction conditions. During friction with the uncoated countersamples and coated (Ti-HIPP and TiN-MS) countersamples, the Sv parameter value during dry friction is higher than during lubrication with S100+ oil. Increasing the oil viscosity caused a decrease in the Sv parameter during friction with the Ti-MS+EPI countersample. For the remaining coated countersamples, the situation was the reverse.

The Sz is the sum of the maximum valley depth Sv and maximum peak height Sp. Strip sample testing with the uncoated countersample, regardless of friction conditions, resulted in a similar increase in the Sz parameter value (Figure 17f). Similarly to the Sp and Sv parameters, changing the oil from S100+ to S300 increases the maximum height Sz.

### 3.3. Temperature

**Figure 21:**
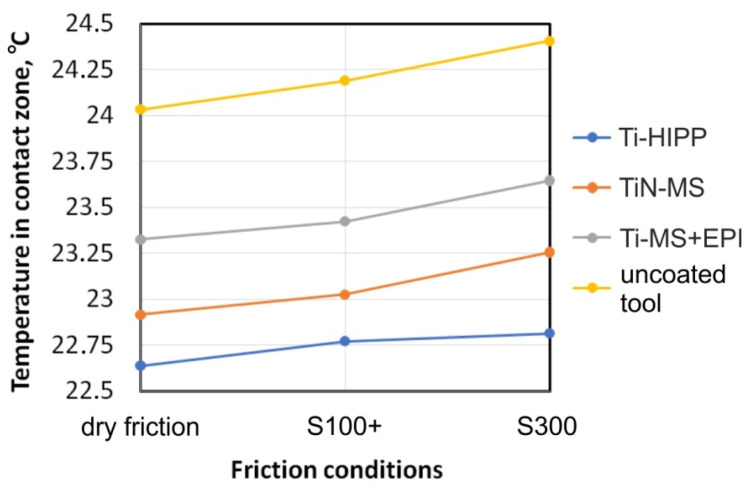
Effect of friction conditions on temperature in contact zone.

The influence of friction conditions on the temperature in the contact zone is shown in Figure 21. During the friction of steel sheet metal against the steel uncoated countersample, the temperature in the contact zone was the highest. This conclusion applies to all friction conditions. Friction with Ti- and TiN-coated countersamples also showed a temperature increase compared to dry friction conditions, but this increase was smaller. The smallest temperature increase was observed for the Ti-HIPP countersample, recorded between 22.6 °C (dry friction) and 22.8 °C (S300 oil lubrication). The magnetron sputtered countersample (TiN-MS) showed a slightly higher temperature increase among the coated countersamples: 22.9 °C (dry friction) to 23.2 °C (S300 oil lubrication). Finally, the highest temperature increase among coated tools was shown by the Ti-MS+EPI-coated countersample 23.3 °C (dry friction) to 23.6 °C (S300 oil lubrication). The applied anti-wear coatings constitute a barrier to heat flow between the strip sample and the countersample substrate. The temperature increase in the contact zone is the result of two phenomena. The first is the frictional interaction of the surface asperities of the sheet metal and the countersample. The second phenomenon is the heating of the sheet metal material as a result of internal friction caused by the stretching of the sample.

In lubricated conditions, the temperature was higher than during dry friction. However, the temperature increase for all types of countersamples was no greater than 0.37 °C. It can be assumed that the lubricant increased the surface of heat transfer, which was transferred in the areas of metallic contact of the surface asperities and through the lubricant located in the valleys of the profile.

## 4. Conclusions

This article presents the results of research on the effect of the type of countersample coating in the BUT test on the coefficient of friction, the change in the sheet metal surface roughness, and the temperature increase in the contact zone. For this purpose, a special device for friction testing was developed and manufactured. The tests were carried out under dry friction conditions and under the conditions of lubrication of the DC01 steel sheet metal surface using two oils with significantly different viscosities. The countersamples with Ti and TiN coatings produced using the high-intensity plasma pulses, magnetron sputtering, and electron pulse irradiation were used. As a result of the conducted experiments, the following main conclusions were obtained:

An increase in the coefficient of friction and sample elongation was observed. Stretching the sample causes a simultaneous change in the topography of the sheet surface and a change in its properties.

The countersamples modified with protective coatings (Ti-HIPP, TiN-MS and Ti-MS+EPI) provided a more stable coefficient value during the entire friction test compared to dry friction conditions.

- During dry friction, the uncoated countersamples caused the largest change in the average roughness Sa.

It was found that during friction with the Ti-MS+EPI countersamples, the kurtosis was most similar to the sheet metal surface in the as-received state.

The skewness Ssk of the sheet metal tested against the coated countersamples was characterized by negative value, which indicates a plateau-like shape of their surface.

Similarly to the Sa and Sku parameters, the friction with the presence of the Ti-MS+EPI countersample provided the most stable Sku value for all friction conditions.

Increasing the oil viscosity caused a decrease in the maximum pit height during friction with the presence of the Ti-MS+EPI countersample.

The highest temperature in the contact zone during friction with all types of countersamples was observed for the uncoated countersample. The temperature increase for all friction conditions was observed for Ti-HIPP, TiN-MS, and Ti-MS+EPI countersamples.

Based on the conducted research, it can be concluded that the Ti-MS+EPI countersample is preferred. It ensured the highest stability of the coefficient of friction in the entire range of sample elongation until fracture. Moreover, during friction with the Ti-MS+EPI coating, the smallest change in the basic sheet surface roughness parameters (Sa, Ssk, and Sku) was observed with the change in friction conditions. The disadvantage of the BUT test is that both front tension force and back tension force take into account the resistance of the material to deformation due to bending and due to frictional contact of the sheet metal surface and the surface of the countersample. Therefore, in the future, tests will be carried out with the use of a freely rotating countersample to separate the friction force from the bending force. During the BUT test, there are variable friction conditions related to the work hardening of the sheet metal material. The constantly increasing contact pressure makes it difficult for the lubricant to effectively separate the rubbing surfaces. Therefore, texturing of the tool surface can be considered, and the size and orientation of the lubricant pockets and the selection of a lubricant with an appropriate viscosity should be the subject of optimization studies.

## Figures and Tables

**Table 1:** Method of preparing countersamples.

Sample Denotation	Description
Ti-HIPP	Ti layer in the processes of the high-intensity plasma pulses (HIPPs), using rod plasma injector (RPI), named IBIS II
TiN-MS	TiN layer in the processes of the magnetron sputtering (MS), using the home-made magnetron device
Ti-MS+EPI	Ti layer in the processes of magnetron sputtering and then electron pulse irradiation (EPI), using the home-made magnetron device and electron gun, named RITM-2M.

**Table 3:** Basic surface roughness parameters of cylindrical countersamples.

Countersample Type	Sq, μm	Ssk	Sku	Sp, μm	Sv, μm	Sz, μm	Sa, μm
146Cr6 (as-received)	2.21	0.568	2.23	6.77	5.25	12.0	1.87
Ti-HIPP	2.11	0.545	1.97	6.50	3.23	9.73	1.81
TiN-MS	2.02	0.515	1.91	4.62	3.28	7.90	1.75
Ti-MS+EPI	1.97	0.237	1.96	6.60	4.79	11.4	1.71

## Data Availability

The raw data supporting the conclusions of this article will be made available by the authors on request.
